# An Investigation of SILC Degradation under Constant Voltage Stress in PDSOI Devices

**DOI:** 10.3390/mi14051084

**Published:** 2023-05-21

**Authors:** Yong Lu, Hongxia Liu

**Affiliations:** Key Laboratory for Wide Band Gap Semiconductor Materials and Devices, School of Microelectronics, Xidian University, Xi’an 710071, China; yonglu_1999@stu.xidian.edu.cn

**Keywords:** stress-induced leakage current, constant voltage stress, trap, partially depleted silicon-on-insulator devices, floating body devices

## Abstract

The stress-induced leakage current (SILC) degradation of partially depleted silicon in insulator (PDSOI) devices under constant voltage stress (CVS) was studied. Firstly, the behaviors of threshold voltage degradation and SILC degradation of H-gate PDSOI devices under constant voltage stress were studied. It was found that both the threshold voltage degradation and SILC degradation of the device are power functions of the stress time, and the linear behavior between SILC degradation and threshold voltage degradation is good. Secondly, the soft breakdown characteristics of the PDSOI devices were studied under CVS. Thirdly, the effects of different gate stresses and different channel lengths on the threshold voltage degradation and SILC degradation of the device were studied. The results showed SILC degradation of the device under positive CVS and SILC degradation of the device under negative CVS. The shorter the channel length of the device was, the greater the SILC degradation of the device was. Finally, the influence of the floating effect on the SILC degradation of the PDSOI devices was studied, and the experimental results showed that the degree of SILC degradation of the floating device was greater than that of the H-type grid body contact PDSOI device. This showed that the floating body effect can exacerbate the SILC degradation of PDSOI devices.

## 1. Introduction

With the continuous reduction in the feature size of MOS devices and the thinning of the gate dielectric thickness, the reliability problem of MOS devices is becoming increasingly serious. In the actual application of MOS devices, they are often affected by external stresses, such as voltage, temperature and irradiation effects. The reliability of the devices are greatly affected under the actions of these external stresses [1,2,3]. Under the action of constant high gate stress, the electrons and holes in the device will obtain higher kinetic energy than that in the thermal equilibrium state, forming thermal carriers. When these thermal carriers obtain enough energy, they can pass through the gate oxide to form a gate tunneling current. These carriers will cause certain damage to the gate oxide in the process of passing through it, resulting in the corresponding oxide trap or interface trap, affecting the gate leakage current of the device. This current is also known as stress-induced leakage current (SILC) [4]. This current increases with the decrease in the thickness of the gate oxide and has become one of the core factors for the continued scaling of flash memory. SILC is also an important parameter used to evaluate the reliability of the gate oxide during the aging test of the device. Thus, the study of SILC is of great significance [5,6,7].

Partially depleted silicon-on-insulator (PDSOI) devices have the advantages of anti-latch-up, a small parasitic effect, fast speed and low power consumption compared with bulk silicon devices and are widely used in electronic communications, automotive electronics, medical, aerospace and other fields, while PDSOI devices have a self-heating effect and floating effect due to the presence of a buried oxygen layer [8,9]. This renders the degradation of PDSOI devices more complex than that of bulk silicon devices under applied stress, which also results in higher requirements for the reliability of the PDSOI device gate dielectric. Therefore, in order to improve the gate medium reliability of PDSOI devices, it is important to study the degradation law of SILC in PDSOI devices.

At present, most of the SILC research has aimed to study bulk silicon devices or MOS capacitors, and there is very little research on the SILC characteristics of ultra-thin gate structure PDSOI devices [10,11,12]. In this paper, the variation law of the threshold voltage degradation and SILC degradation of PDSOI devices with the stress time under constant voltage stress (CVS) is studied, together with the effects of positive and negative CVS and different device channel lengths on the SILC degradation of PDSOI devices and, finally, the influence of the floating effect on the SILC degradation of PDSOI devices.

## 2. Materials and Methods

**Figure 1:**
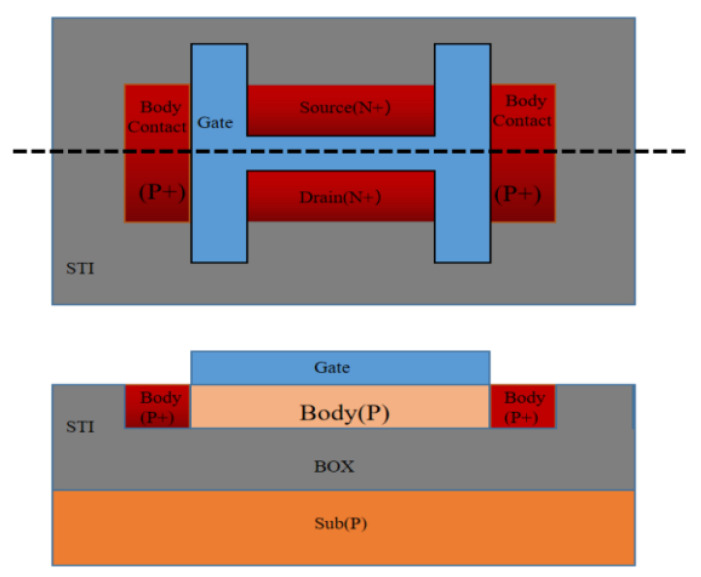
Schematic diagram of the structure of the H-gate PDSOI device.

In the experiment, an H-gate PDSOI device and floating body device manufactured through the 0.13 μm PDSOI process were used. Figure 1 is a schematic diagram of the device structure, in which the silicon film thickness of the top layer of the device is 100 nm, the thickness of the gate oxide (SiO_2_) is 2 nm, and the thickness of the buried oxygen layer is 145 nm. The device dimensions and stress conditions used in the experiment are shown in Table 1.

The CVS stress experiments were conducted with an Agilent B1500 semiconductor parameter analyzer using the quasi-DC Stress–Measure–Stress (SMS) technique [13]. CVS measurement includes the application of a voltage stress higher than the operating voltage to the gate in order to accelerate degradation. The source, drain, and substrate contacts were grounded in this experiment.

In this method, the stress gate voltage is set to 3.7 V, the total stress time is set to 1000 s, the temperature is set to 25 °C, the stress is interrupted immediately after the pre-set stress time is reached, and the electrical characteristics of the device after the application of stress are tested in time, including the transfer characteristics of the device and the gate current characteristics. Among them, the test condition of the device transfer characteristic is the fixed leakage voltage, set to 0.1 V, while the source and body terminals are grounded, the gate voltage is scanned from −0.5 V to 1.2 V, the drain current I_d_ data are collected for storage, and the value of the device threshold voltage is extracted from the transfer curve using the maximum transconductance method. Regarding the test conditions of the gate current, the drain, source and body terminals are grounded, the drain voltage V_g_ is scanned from 0 V to 1.2 V, and the gate current I_g_ data are collected for storage.

## 3. Results

### 3.1. The I–V Characteristic Degradation of the PDSOI Device under CVS

#### 3.1.1. The Degradation of Transfer Characteristics under CVS

**Figure 2:**
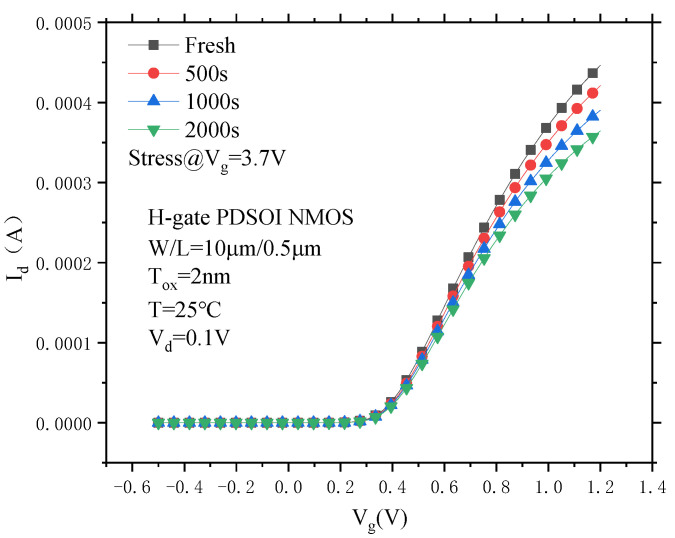
The degradation of the transfer characteristics of the PDSOI device under CVS.

Figure 2 shows the degradation curve of the device transfer characteristics under CVS, and it can be seen that after the application of CVS, the drain current in the linear region of the PDSOI NMOS device decreases, and the threshold voltage is forward-drifted.

In order to more intuitively understand the degradation of the PDSOI device threshold voltage and the saturation drain current in the linear region under CVS, the corresponding parameters were extracted from Figure 2, and the degradation percentage was calculated. The results are shown in Table 2. According to the calculation, after 2000 s of CVS, the saturation drain current in the linear region changes from 451.9 μA to 364.37 μA, the drift amount ΔI_din_ is 87.53 μA, the degradation percentage is 19.37%, the device threshold voltage changes from 356.829 mV to 375.460 mV, the drift amount ΔV_th_ is 15.63 mV, and the degradation percentage is 4.38%.

For a typical MOS device, the threshold voltage equation can be expressed as:

(1) $$V_{th}=2\phi_{F}+\frac{\sqrt{2q\varepsilon_{s}\varepsilon_{o}NA\left(2\phi_{F}\right)}}{C_{ox}}+\phi_{MS}-\frac{Q_{ox}}{C_{ox}}-\frac{Q_{it}\left(\phi_{s}=2\phi_{F}\right)}{C_{ox}}$$

In this formula, $\phi_{F}$ is the Fermi potential, $\varepsilon_{o}$ is the vacuum permittivity, $\varepsilon_{s}$ is the permittivity of SiO_2_, *q* is the unit charge, *N_A_* is the doping concentration of the substrate, $\phi_{MS}$ is the difference in work function between the metal and the semiconductor, *C_ox_* is the oxide layer capacitance, *Q_it_* is the interface trap charge density at the Si/SiO_2_ interface, and *Q_ox_* is the charge density in the oxide layer. From the formula, it can be seen that the threshold voltage degradation is related to interface traps and oxide traps.

**Figure 3:**
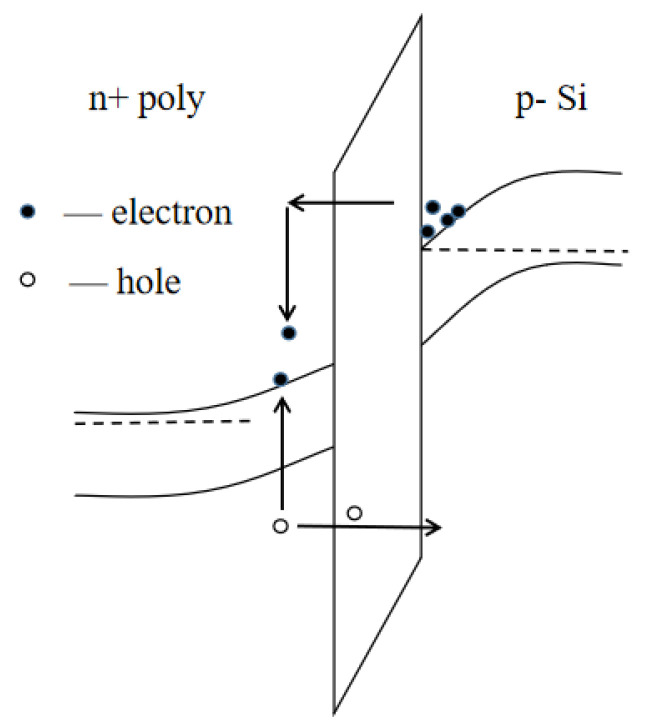
The schematic diagram of carrier transport under CVS.

**Figure 4:**
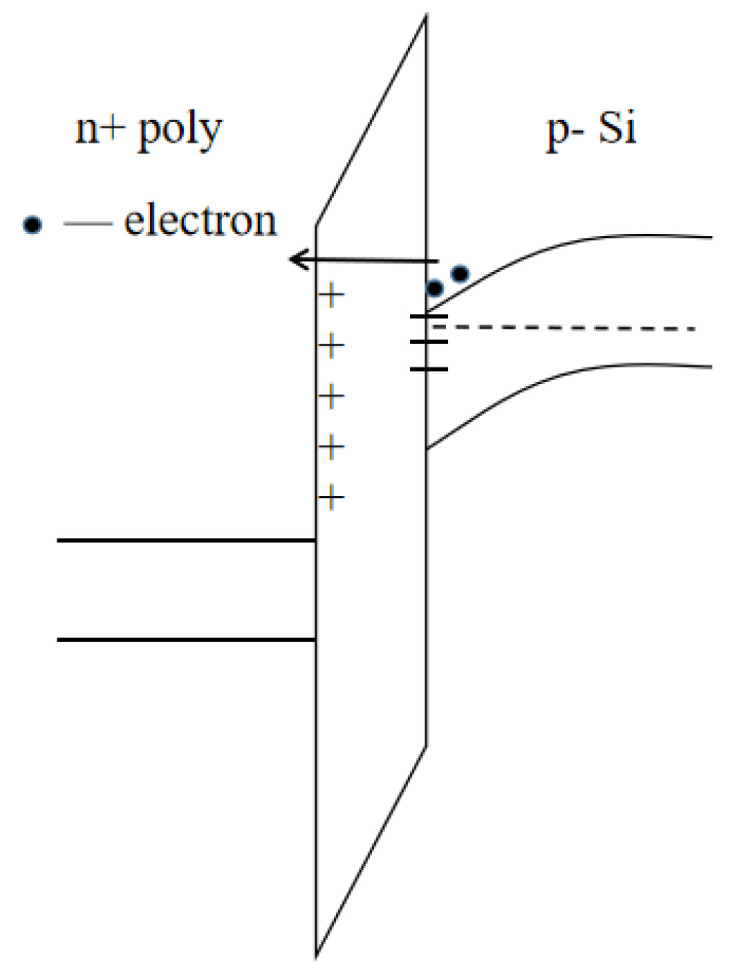
The distribution of the positive charge of traps in the oxide layer “+” and interfacial traps “−” in the oxide.

Figure 3 is a schematic diagram of the internal carrier transport process of the device under CVS, and Figure 4 is a schematic diagram of the distribution of oxide traps and interface traps on the device gate oxide. 

Under CVS, the reverse layer channel electrons of the PDSOI device move towards the polysilicon gate under the acceleration of a strong electric field and obtain energy from it. Upon reaching the gate, the electrons and the polysilicon gate lattice undergo collision ionization to produce a large number of electron–hole pairs. The holes generated at the gate under the action of the electric field tunnel go back to the oxide layer and move towards the substrate. Near the interface between the gate and the gate oxide, part of the hole is trapped by the oxide trap, forming a trap positive charge. The trap positive charge will lead to negative drift of the device threshold voltage. When high-energy electrons pass through the gate oxide, the Si-H bond and Si-O bond at the Si/SiO_2_ interface will be broken to form a hanging bond, resulting in interface traps. The interface traps will trap the negative electrons and cause the device threshold voltage to drift positively. According to the final experimental results, the threshold voltage of the PDSOI device is positively drifted, which indicates that the interface trap dominates V_th_ degradation.

#### 3.1.2. The Degradation of the Gate Current under CVS

**Figure 5:**
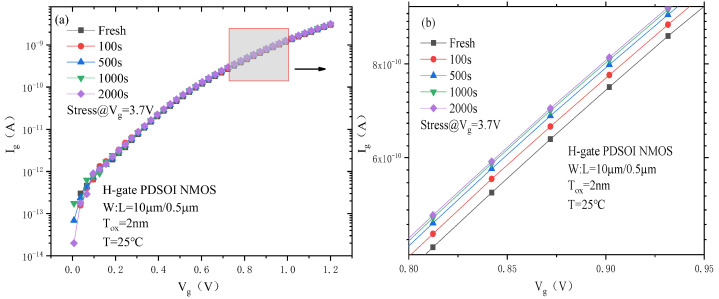
The gate current change curves of the PDSOI device under CVS. (**a**) Complete, (**b**) Enlarged.

Figure 5 shows the gate current degradation curve of the PDSOI NMOS device under CVS, and because the gate current is of the order of nA, the change in the gate current under CVS is not obvious. In order to more intuitively understand the degradation of the device gate current under CVS, the gate current I_g_ at the test gate voltage of 1.2 V was extracted from the figure, and the degradation percentage was calculated. The results are shown in Table 3. From the table, we can see that the device gate current increases with the increase in the stress time under CVS, and the gate current degrades by 6.75% after the device is subjected to 2000 s of CVS.

The increase in the gate current is also related to oxide traps and interface traps [14]. According to the trap-assisted tunneling theory, the trap in the oxide layer will trap holes to form trap positive charges. The presence of the trap positive charges will enhance the local electric field in the gate oxide layer and reduce the barrier height of the gate oxide layer. The electron tunneling probability increases with the enhancement of the local electric field in the gate oxide layer and the decrease in the barrier height, which ultimately leads to the increase in the gate tunneling current of the device. The existence of the interface trap can be regarded as the transition energy level in the electron tunneling process. When testing the device gate current, the device is in an inverted state, and at this time, the interface trap will capture part of the channel electrons. The captured channel electrons have a certain probability, based on the trap energy level, of continuing to tunnel towards the gate, equivalent to the existence of an interface trap, which becomes a “springboard” in the entire tunneling process of the electrons. The increase in the interface trap will increase the probability of electron tunneling, so that the gate tunneling current increases [15,16].

### 3.2. Threshold Voltage Degradation and SILC Degradation in Relation to the Stress Time

**Figure 6:**
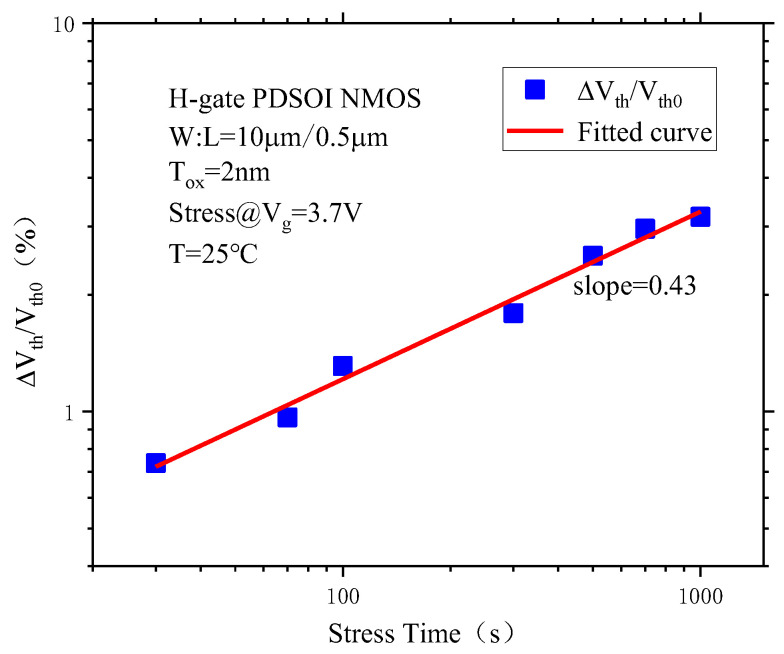
The threshold voltage degradation of the PDSOI NMOS device Δ*V_th_/V_th0_* varies with the stress time under CVS.

Figure 6 shows the threshold voltage degradation of the PDSOI NMOS devices Δ*V_th_/V_th_*_0_ as a function of the stress time after CVS. It can be seen from the figure that in the bilogarithmic coordinates, there is a linear behavior between the threshold voltage degradation amount Δ*V_th_/V_th_*_0_ and the stress time, which shows that the threshold voltage degradation and the stress time obey the power rate behavior. The behavior between the two can be expressed as:

(2) $$\mathrm{lg}(\Delta V_{th})=A+n\mathrm{lg}t$$

Its exponential expression is:

(3) $$\Delta V_{th}=10^{A}t^{n}$$

Above, *A* is the constant coefficient, and *n* is the time acceleration coefficient, which represents the slope of the fitted curve in the bilogarithmic coordinates, and the slope of the curve in the bilogarithmic coordinates is approximately 0.43, that is, *n* = 0.43.

**Figure 7:**
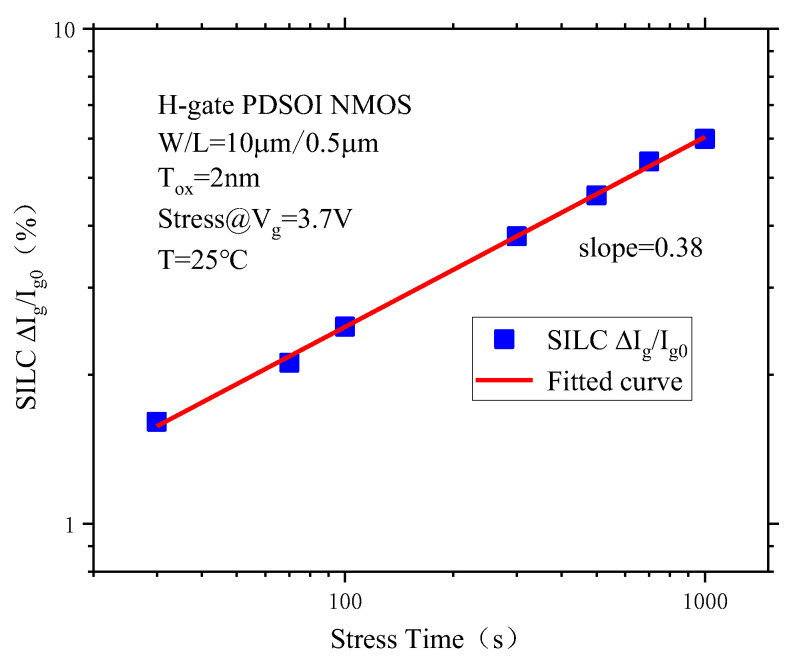
The SILC degradation of PDSOI devices varies with the stress time under CVS.

SILC can be defined as (I_g_ − I_g0_)/I_g0_, where I_g0_ is the initial gate current before stress is applied to the device, and Ig is the device gate current after stress. Figure 7 shows the SILC degradation curve of the PDSOI NMOS device with the stress time after CVS. It can be seen from the figure that the SILC degradation and stress time also show a good linear behavior in the bilogarithmic coordinates, which means that SILC and the stress time also obey the power rate behavior. Thus, the functional behavior between SILC and the stress time can be expressed as:

(4) lg(SILC)=B+αlgt

Its exponential expression is:

(5) $$SILC=10^{B}t^{\alpha }$$

Here, *B* is the constant coefficient, which is the time acceleration coefficient that represents the slope of the fitted curve in the bilogarithmic coordinates, and the slope of the curve in the bilogarithmic coordinates is approximately 0.38, that is *α* = 0.38.

**Figure 8:**
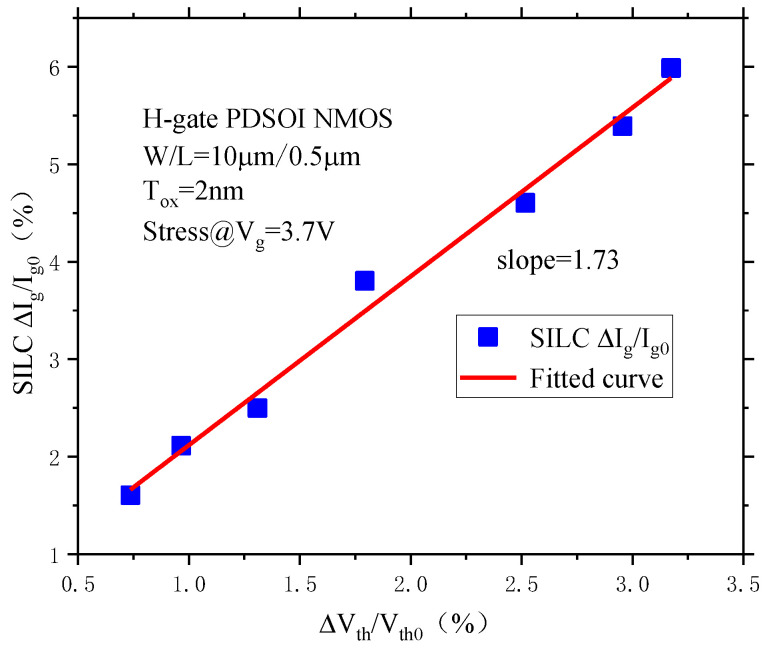
Behavior between threshold voltage degradation and SILC degradation.

The degradation form of SILC under constant voltage stress is very similar to the degradation form of the threshold voltage. Thus, we compared the degradation of SILC under constant voltage stress with the degradation of the threshold voltage, and the results are shown in Figure 8. From the figure, we can see that the degradation of SILC and the degradation of the threshold voltage show a good linear behavior, and the linear fitting parameter is 1.73.

The drift of the device threshold voltage is due to the increase in oxide traps and interface traps, whereas the increase in the gate current is also related to the increase in oxide traps and interface traps. This means that the SILC degradation represents the accumulation of oxide traps and interface traps on the device gate oxide [17], which can be used to characterize the reliability degradation of the device gate oxide layer.

### 3.3. Soft Breakdown of PDSOI Devices under CVS

For MOS devices, the breakdown of the gate oxide can generally be divided into two types. One is intrinsic breakdown caused by electron collision ionization. The second is where the local area current surge of the gate oxide causes the temperature of the gate oxide to rise rapidly, causing SiO_2_ to melt and induce thermal breakdown. When the gate oxide thickness is less than 5 nm, a new breakdown mechanism affects the MOS device, namely, soft breakdown, also known as pre-breakdown or quasi-breakdown. In the process of the constant voltage stress experiments, we found that when the gate oxide (SiO_2_) thickness of the PDSOI device was 2 nm, soft breakdown of the device gate oxide occurred.

**Figure 9:**
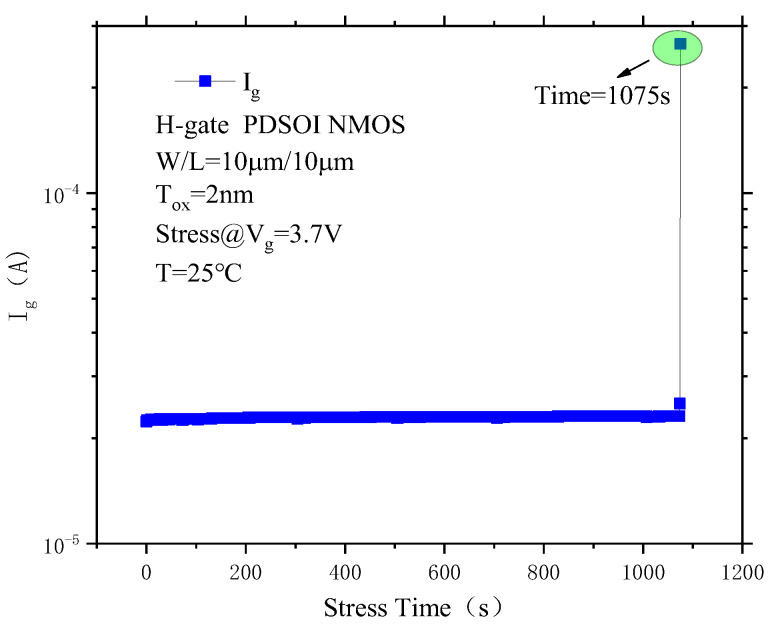
Breakdown curve of a PDSOI device under CVS.

Figure 9 shows the gate current as a function of stress time during CVS. It can be seen from the figure that the device gate current change is not obvious at the beginning of the stress test, but the device gate current increases sharply at T = 1075 s, which indicates that the device breaks down at this time. At this point, the gate stress is immediately removed, and the electrical characteristics of the device are tested, including the device gate current, output characteristics, and transfer characteristics.

**Figure 10:**
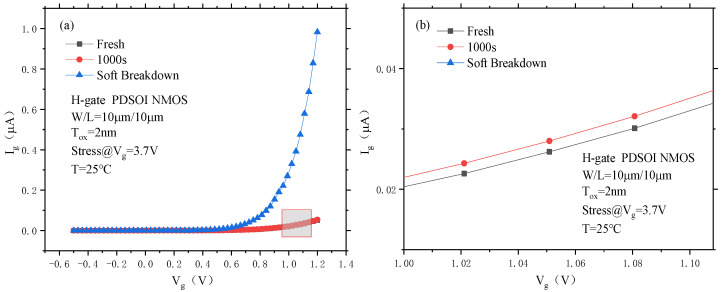
Gate current curve of the PDSOI device before and after soft breakdown under CVS. (**a**) Complete, (**b**) Enlarged.

Figure 10 shows the results of the gate current test after stress removal from the PDSOI device. From the figure, it can be seen that the gate current of the PDSOI device after soft breakdown reaches 0.983 μA at a gate voltage of 1.2 V. Compared with the initial gate current of 0.051 μA, the gate current of the device after soft breakdown increases by approximately 20 times. After soft breakdown, the device has an approximate exponential behavior between the gate current and the gate voltage.

**Figure 11:**
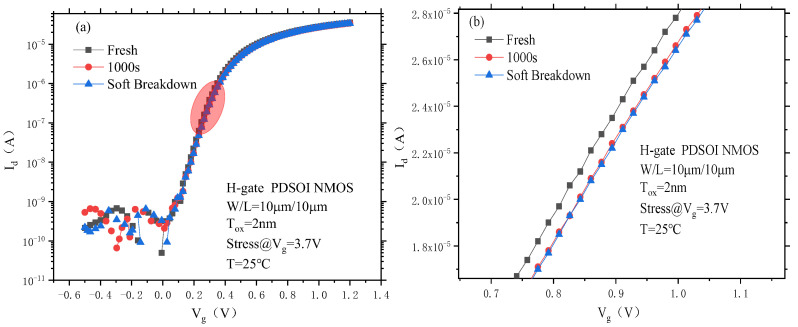
Transfer characteristic curve of the PDSOI device before and after soft breakdown under CVS. (**a**) Complete, (**b**) Enlarged.

Figure 11 shows the PDSOI device transfer characteristic change curve before and after soft breakdown. It can be seen from the figure that the off-state leakage current of the device under a negative gate voltage before and after the soft breakdown remains at 10^−10^ to 10^−9^A. The overall curve drifts to the right along the transverse coordinate axis, which indicates that the soft breakdown does not have a great impact on the switching characteristics of the device, and the device does not fail completely at this time.

**Figure 12:**
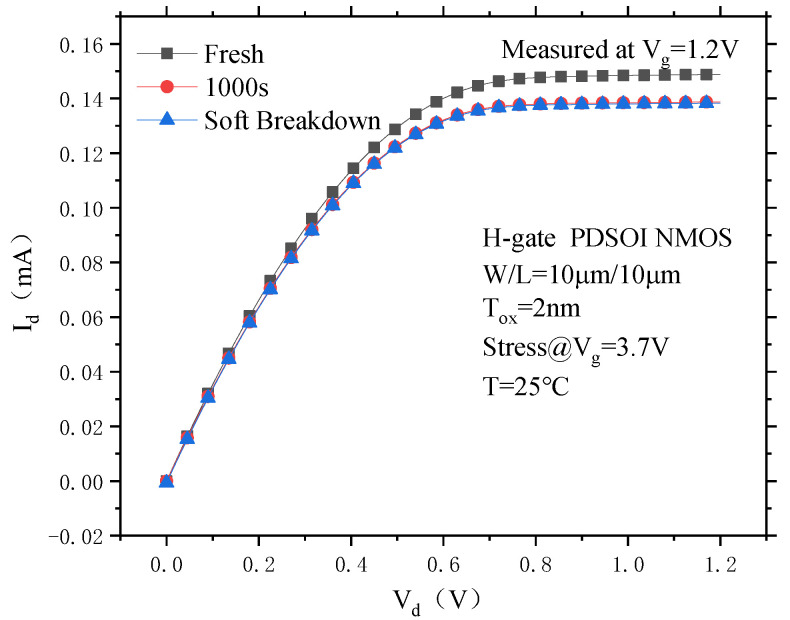
Transfer output characteristic curve of the PDSOI device before and after soft breakdown under CVS.

Figure 12 shows the output characteristic change curve of the PDSOI device before and after soft breakdown. It can be seen from the figure that when the test gate voltage is 1.2 V, the output current of the device drops after soft breakdown, and the output current at 1000 s is slightly greater than the output current after soft breakdown. This is due to the electrons trapped by the interface trap that are generated during the CVS process, scattering the channel carriers and resulting in a decrease in the channel carrier mobility and a decrease in the device output current. However, the degradation of the output current at 1000 s is not significantly different from the degradation of the output current after soft breakdown, which indicates that the number of interface traps of the PDSOI device does not increases much during the period from 1000 s to soft breakdown.

From the above experimental results, it can be seen that the switching characteristics of the PDSOI device are basically unchanged after soft breakdown, but the gate leakage of the device increases sharply. This also means that when performing accelerated stress testing of a device, a focusing on the output characteristics or transfer characteristics of the device alone may not be sufficient to detect the presence of soft breakdown. We need to pay attention to both the change in the gate current and the SILC during stress so as to better analyze the degradation of the device gate medium reliability. In particular, when life prediction is carried out for ultra-thin gate oxide (T_ox_ = 2 nm) PDSOI devices through Time-Dependent Dielectric Breakdown (TDDB) testing, the lifetime of PDSOI devices can be overestimated due to soft breakdown. Therefore, SILC can be used in TDDB testing to monitor the soft breakdown time of the device.

### 3.4. PDSOI Device SILC Degradation under Different Conditions

#### 3.4.1. Effects of Positive and Negative Gate Voltages on the SILC Degradation of PDSOI Devices

**Figure 13:**
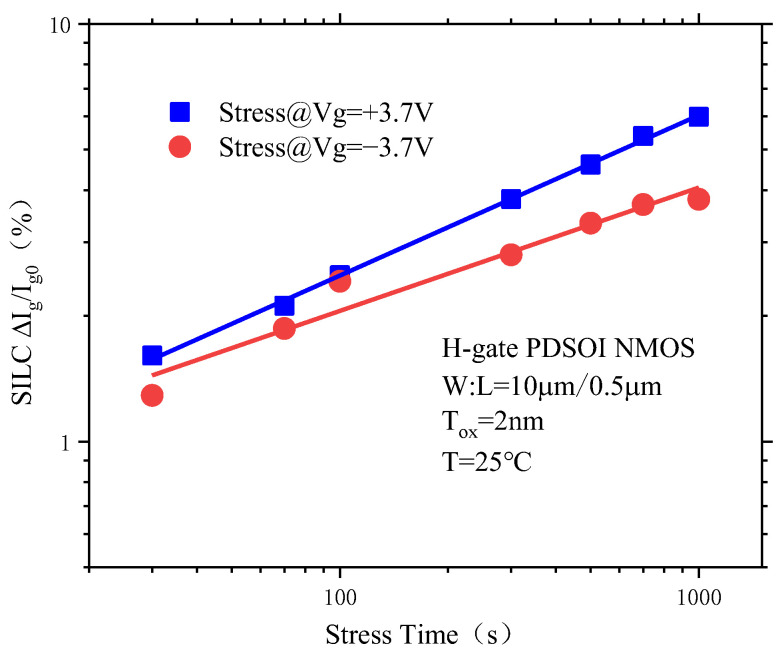
SILC degradation of the PDSOI devices under positive and negative CVS.

Figure 13 shows the SILC degradation curve of PDSOI devices under positive and negative CVS. From the figure, it can be seen that the SILC degradation of PDSOI devices under positive CVS is greater than that of SILC degradation under negative CVS. In the positive CVS process, electrons undergo collision ionization at the polysilicon gate, whereas in the negative CVS process, electrons undergo collision ionization at the Si/SiO_2_ interface, which plays a positive role in the generation of interface traps. However, holes have a chance to fall into the Si/SiO_2_ interface, which will offset the impact of some interface traps on the device. Therefore, the SILC degradation of PDSOI devices under positive CVS is greater than SILC degradation under negative CVS.

#### 3.4.2. Effect of Channel Length on the SILC Degradation of PDSOI Devices

**Figure 14:**
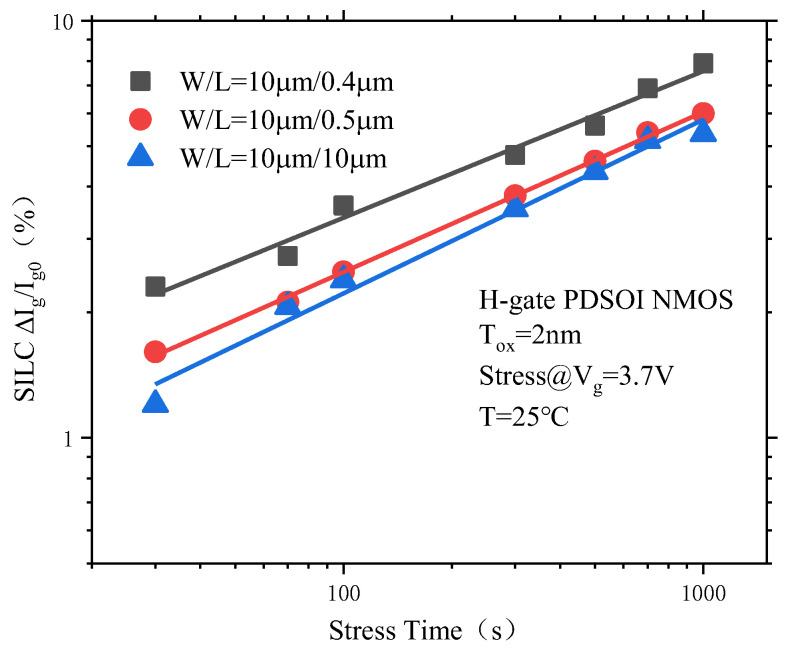
PDSOI device SILC degradation at different channel lengths.

Figure 14 shows the SILC degradation curve of the PDSOI device with the stress time under different channel lengths. It can be seen that the degree of SILC degradation of the PDSOI devices increases with the increase in the channel length. This is because under CVS, only a gate voltage bias applies to the gate of the device, and the rest of the ends are grounded. The longitudinal electric field of the device gate oxide can be considered to be uniformly distributed. The generated trap damage to the gate oxide is also approximately evenly distributed along the channel. The shorter the channel length is, the greater the proportion of trap damage to the transverse channel will be, which also means that the SILC degradation of the device will be more serious.

#### 3.4.3. The Floating Effect on the SILC Degradation of PDSOI Devices

**Figure 15:**
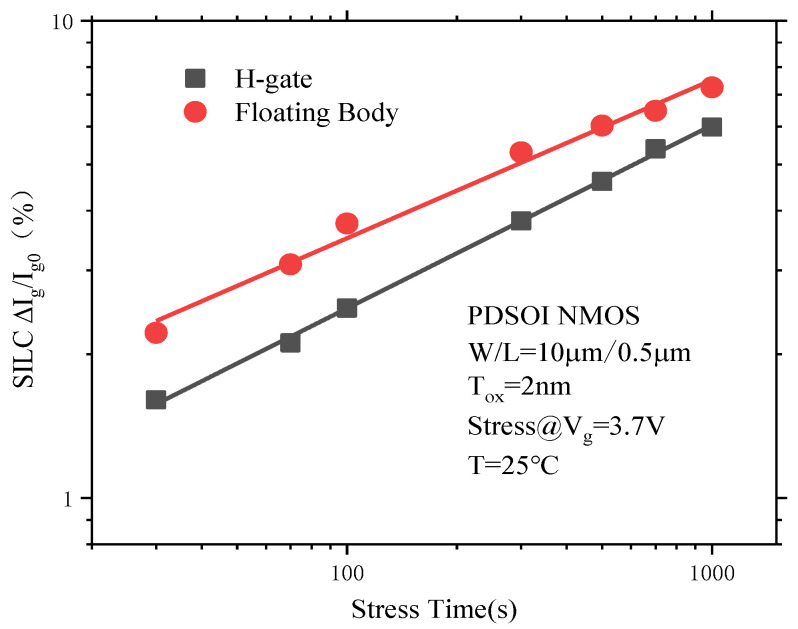
The SILC degradation of the floating device and the H-gate contact device change curves with the stress time.

Figure 15 shows the SILC degradation of the floating device and the H-type gate contact device with the change curve of the stress time. It can be seen from the figure that under the same stress time, the SILC degradation of the floating device is greater than that of the H-gate contact device, because the body region of the PDSOI floating device is in a suspended state. A proportion of the holes generated via collision ionization under the action of CVS accumulate in the body region of the floating device, resulting in an increase in the body potential, a decrease in the device threshold voltage, and an increase in the electric field of the device gate. This will ultimately lead to the intensification of the SILC degradation of the floating device [18].

## 4. Conclusions

In this paper, the SILC degradation of PDSOI devices was investigated. The degradation of the threshold voltage and gate current of PDSOI devices under CVS and their behavior with respect to stress time were studied. The experimental results show that under CVS, the threshold voltage degradation and SILC degradation of PDSOI devices show a power function behavior, and the threshold voltage degradation and SILC degradation originate from the increase in the number of oxide traps and interface traps. This means that SILC can be used to characterize the reliability of the SILC gate oxide of the device. The soft breakdown characteristics of PDSOI devices were studied under CVS. The SILC degradation of PDSOI devices was studied under different conditions, and the results showed that the SILC degradation of PDSOI devices under positive CVS was greater than that of PDSOI devices under negative CVS. The smaller the channel length of the PDSOI device was, the more severe the SILC degradation was. The floating effect exacerbates the SILC degradation of PDSOI devices.

## Figures and Tables

**Table 1:** Devices used in the experiment and stress test conditions.

Device	Body Contact	Width–Length Ratio (W/L)	Constant Voltage Stress (V_g_)
PDSOI NMOS	H-gate	10 μm/0.4 μm	+3.7 V
		10 μm/0.5 μm	
		10 μm/10 μm	
		10 μm/0.5 μm	−3.7 V
	Floating body	10 μm/0.5 μm	+3.7 V

**Table 2:** The V_th_ degradation and I_din_ degradation of the PDSOI device under CVS.

Stress Time (s)	V_th_ (mV)	ΔV_th_ (%)	I_din_ (μA)	ΔI_din_ (%)
0	356.829	0	451.90	0
500	365.813	2.518	421.08	7.23
1000	368.156	3.174	390.41	13.61
2000	375.460	4.38	364.37	19.37

**Table 3:** The change in gate current measured at V_g_ = 1.2 V.

Stress Time (s)	I_g_ (nA)	ΔI_g_ (%)
0	2.96	0
100	3.04	2.71
500	3.10	4.73
1000	3.14	6.08
2000	3.16	6.75
